# Dynamic Response of Components Containing Polymer Composites in the Resonance Region for Vibration Amplitudes up to 5g

**DOI:** 10.3390/polym14225051

**Published:** 2022-11-21

**Authors:** Zuzana Murčinková, Pavel Adamčík, Dominik Sabol

**Affiliations:** 1 Department of Design and Monitoring of Technical Systems, Faculty of Manufacturing Technologies with a Seat in Prešov, Technical University of Košice, Bayerova 1, 080 01 Prešov, Slovakia; 2 Technická Diagnostika, Ltd., Jilemnického 3, 080 01 Prešov, Slovakia

**Keywords:** native design, rotational speed, additional material, passive damper, polymer composite, filler, vibration amplitude reduction, mass

## Abstract

This paper focuses on high-speed-operation textile machines with the aim of increasing the rotational speed by operating within the resonance region to vibration amplitudes up to 5g. The native design does not allow keeping the vibration amplitude under 5g, which is a safe operation mode, for revolutions more than 120,000 min^−1^. The innovative modification of the design was made by the incorporation of polymer composite materials with carbon dust, glass hollow microspheres, and silica sand fillers to the rotor-bearing casing; moreover, through the incorporation of a multilayered foam composite structure and particle damper to the pressure plate of the mechanical machine system. By using the approach of supplementing with high-damping composites, the existing native design can be used, thus avoiding the costly production of new components and subassemblies with modified shapes and dimensions. Twelve possible combinations of mentioned modifications were tested, evaluated and compared with the native design made of steel, as standard structure material in mechanical engineering. The average vibration amplitudes were evaluated in the region before the resonance peak and in the range of the resonance peak, i.e., 120,000–135,000 min^−1^. Significant vibration amplitude reductions in the range from 30 to 70% of the average vibration amplitude were obtained. The vibration amplitude reduction results were evaluated considering the mass through the amplitude reduction efficiency coefficient.

## 1. Introduction

The dynamic behaviour of machine tools is directly related to the materials used in their construction. Modern machines need to be lightweight and provide high-speed performance, in our case, up to 2250 Hz. The principal parameters influencing the dynamic response and sound transmission are mass, stiffness and damping. An unwanted dynamic response is a resonance phenomenon manifesting through large vibration amplitude values. Resonance occurs when the driving frequency of the forcing dynamic load is the same as the natural frequency of the mechanical system. Low damping makes the amplitude at the resonance peak several times larger than static displacement. In case the forcing load frequency is not able to change, the first choice for vibration control is resonance avoidance by tuning the dimensions of critical components so that their natural frequencies do not coincide with engine order lines at running speeds [1].

There is a mechanical–physical principle that means that the region of the resonance peak is the damping-controlled region. Therefore, larger damping results in reducing the vibration amplitudes in the resonance region. The displacement response *x* of a damped mass-spring system is defined at frequency conditions as follows [2]:

(1) $$x=\left(\frac{F_{0}}{k}\right)\sin\omega t\;\;\;\mathrm{for}\;\omega <<\omega_{n}$$

(2) $$x=\left(\frac{F_{0}}{2k\zeta }\right)\sin\left(\omega_{n}t+\frac{\pi }{2}\right)\;\;\;\mathrm{for}\;\omega =\omega_{n}\;\mathrm{resonance}\;\mathrm{region}$$

(3) $$x=\left(\frac{F_{0}}{m\omega^{2}}\right)\sin\omega t\;\;\;\mathrm{for}\;\omega >>\omega_{n}$$

where *ω* is the frequency of excitation, *ω***_n_** is the natural frequency of an undamped system, *F*_0_ is the amplitude of applied force, *k* is the stiffness, *t* is time, *ζ* is the damping ratio, *m* is the mass. According to above three frequency conditions, the vibrating mechanical system is described as stiffness-controlled, damping controlled, and mass-controlled, respectively, depending on which element is primarily responsible for the system behavior. The damped natural frequency *ω***_d_** is defined as follows:

(4) $$\omega_{d}=\omega_{n}\sqrt{1-\zeta^{2}}$$

The good vibration isolator is of low transmissibility, whereas it transmits a low amount of force or motion. The relation of the transmitted force *F*_T_ to the applied force *F*_0_ can be expressed in terms of transmissibility *T* as follows:

(5) $$T=\sqrt{\frac{1+{\left(2\zeta \omega /\omega_{n}\right)}^{2}}{{\left(1-\omega^{2}/\omega_{n}^{2}\right)}^{2}+{\left(2\zeta \omega /\omega_{n}\right)}^{2}}}$$

The fact that the resonance peak is the damping-controlled region was principal to solving the problem described in this paper. Moreover, the increased damping results in reduced dynamic stresses, short decay of free vibrations and an increase in sound transmission loss above the critical frequency [3]. Vibration control can be achieved by passive or active dampers. Passive vibration control involves the modification of stiffness, mass and or damping of the vibrating system to make the system less responsive to its vibrating environment [4]. Damping causes the dissipation of vibration energy into heat energy, dissipated by an appropriate material with a high dissipation energy ability. Previous decades in material research have been focused on advanced materials with an inner structure characterized by high damping. Various types of composite materials and hybrids have allowed high structural damping to be achieved in production machinery. The aluminum foam sandwiches and carbon fiber reinforced polymer ram showed a damping that is about 20 and 3 times, respectively, greater than the conventional steel ram [5]. The results of the experimental study into the effect of improving the damping ratio revealed that the addition of steel by a particulate-reinforced polymer composite (25%, 50%, 75% of volume of hybrid structure) showed a high damping ratio and increased the damping of the structure by a large amount (by 6.5, 40 and 80 times) with the reduction in stiffness (30%, 50%, 70%) when it was applied to the grinding machine [6].

Polymer composites are materials with very good vibration and sound damping. However, practical applications often need high mechanical properties such as tensile and compression strength, water adsorption and resistance, destruction mechanisms as tested by the authors in [7,8], dynamic compression strength, deformation, and toughness as tested by the authors in [9] and, at the same time, a high damping capacity. These are incompatible properties for metals as single monolithic materials. However, metal matrix composites allow the mentioned properties to be combined [10]. Moreover, to dissipate vibration energy, the materials can form layered configurations, such as free [11] and constrained layer damping applications with viscoelastic materials [12], sandwich plates and functionally graded plates [13]. To maximize the effect of vibration damping, the geometry parameters of the inner structure of multiphase materials, such as composites, or architected cellular materials are optimized. Thus, the performance of optimized multiphase micro-architected materials is far superior to those of the individual solid phases [14]. The mentioned advanced materials guarantee a high damping capacity along with low weight. The amplitude reduction coefficient shows the relationship between amplitude reduction and the additional mass of the material acting as a passive damper [15].

The different factors including excitation frequency, number of material layers, material thickness, density and inertial mass influence the displacement transmissibility of multilayered composite structures [16]. For polymers, temperature- and frequency-dependent damping is typical.

One of the presented design modifications incorporates particle damping using lead particles freely moving in cavities, an effective passive vibration-control technology, which is a new trend at the present stage. Particle dampers are devices that work by a combination of impact and friction damping of highly nonlinear behavior [17]. Vibration energy is dissipated by the damper through inelastic collisions and friction between particles [18]. As the primary structure vibrates, kinetic energy is significantly absorbed through the combined effects of particle-to-particle and particle-to-wall inelastic collisions and frictional losses, producing damping of the primary structures [19]. Previous experimental results have shown that particle damper can reduce the resonance of the primary structure by more than 50% under sinusoidal or random excitation [20]. Applications of particle damping inside cavities of gear transmission systems and the investigation of the effects of the particle radius, coefficient of friction and restitution coefficient on the dynamic characteristics were explored in [21].

Many articles have presented analyses of the properties of layered composite materials, mainly simulations, but also experimental measurements. The number of viscoelastic damping material-related research articles increased from 480 publications in 2000 to 1850 in 2014 on Elsevier Village, as reported in [22]. However, there is much less literature concerning specific applications in mechanical engineering, to which our presented paper contributes. Nevertheless, the applications in mechanical engineering using the damping properties of layered damping composites include, for example, the improvement of the damping capacity of the headstock of the high-speed precision horizontal machining center by a constrained layer damping structure adhered to the surface of the headstock, as presented in [23], and the space station truss structure with long tubes, with a viscoelastic damping layer for vibration absorption, as presented in [24]. For the field of application of industrial printing machines, hybrid aluminum/composite co-cured tubes with a viscoelastic interface layer were developed by the authors in [25].

Experimental studies have been mainly based on experimental modal analysis, measurement of vibrations and analysis of the dynamic signal, acoustic emission analysis, temperature measurements, etc., with the goal of finding correlation data between testing, and simulation results and obtaining the optimized structure. Many other experimental studies or the virtual testing of machine tools and their frames and modules, e.g., automatic billet straightening machine [26], gear transmissions [27], and production workplace [28] with the aid of the finite element numerical method [29], multiscale modelling using the finite element method [30] and other numerical methods intended for polymer composites have been described by the authors in [31,32], as well as with a stochastic approach by the authors in [33].

The presented study focuses on two components of the mechanical system and adding the polymer particle composites with three fillings, layered structured composites and a particle damping technique. The addition of the mentioned high-damping-capacity materials to the mechanical machine system does not require high costs compared to those required for a shape and dimension change in casting components.

## 2. Experimental Structure and Scheme

**Figure 1:**
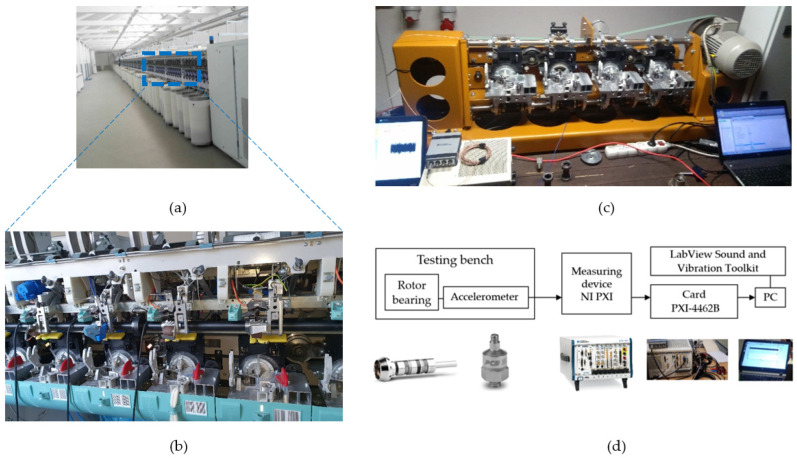
(**a**) Textile machine line, (**b**) Part of textile machine with spinning units, (**c**) Testing bench during measuring, (**d**) Measuring chain.

In the textile machine mechanical systems intended for fibre spinning presented in this paper, the increasing operating rotational speed for economic reasons came up to the resonance region. The goal of developing the new generation of textile machine line (Figure 1a,b) was an increase in speed from the current state of 105,000–110,000 min^−1^ to the planned speed of 130,000–135,000 min^−1^. The higher rotational speed is reflected in the larger amount of the produced spun fibre, but also in high amplitudes of rotor-bearing vibrations that cause a decrease in the time-life of the rotor bearings from the required 20,000 h to 5000–7000 h. Therefore, the reduction of vibration amplitudes is crucial. The modification of components dimensions was not the approach applied to achieve the goal.

The experimental study was carried out on the testing bench (Figure 1c). Absolute vibrations were measured in accordance with standard ISO 10816-3: Mechanical vibrations (https://www.iso.org/standard/50528.html, accessed on 7 September 2022). The limits of the measured amplitude values, 5g and 10g, were estimated using long-term testing based on the lifetime test, i.e., the condition for testing is the required lifetime of 8000 h. After that time, 93% of the rotors should be satisfying rotors, 7% should not. The testing batch for the mentioned lifetime test was approximately 2500 rotors. According to the condition, the ranges of up to 5g, up to 10g, and over 10g, represent Safe operation, Warning (alarm 1), and Danger (alarm 2), respectively.

The measurement setup involved the following (Figure 1d):

− An accelerometer PCB model 352A60 (PCB Piezotronics, Depew, NY, USA), with a frequency range of up to 65 kHz and a sensitivity of 10 mV/g;
− Dynamic Signal Acquisition device PXI-4462, a NI PXI Sound and Vibration Module Meter (National Instruments Corporation, Austin, TX, USA);
− A/D converter resolution 24 bits, sample rates, samples-per-second 1 kS/s to 204.8 kS/s in 181.9 μS/s increments;
− Software for advanced analysis of the dynamic signal base using LabView Sound and Vibration Toolkit software (National Instruments Corporation, Austin, TX, USA).

**Figure 2:**
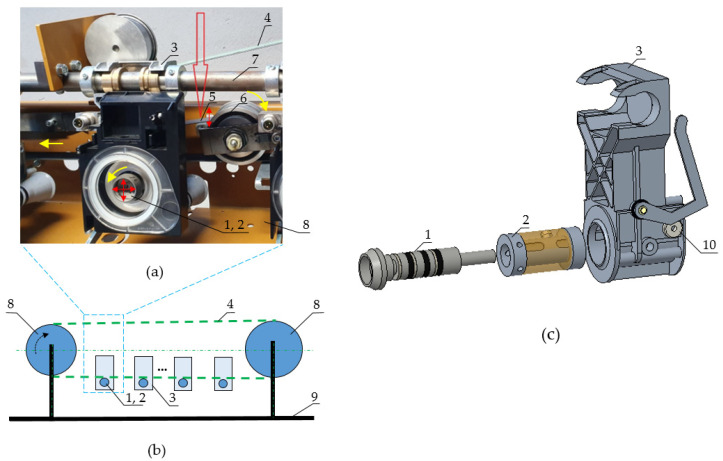
(**a**) Spinning unit of testing bench, (**b**) Schema of testing bench, (**c**) Decomposition of spinning unit, additional polymer composite material (transparency brown)—rotor bearing (1), rotor casing (2), housing body (3), driving belt (4), pressure plate (5), idler pulley (6), rod (7), driving and driven pulley (8), frame (9), accelerometer (10).

One spinning unit of the testing bench representing the spinning unit of the textile machine line is visible in Figure 2a, with a schema of a testing bench in Figure 2b. The placement of the rotor bearing (1) in the rotor casing (2) and the housing body (3) is shown in the subassembly in Figure 2c, along with the accelerometer (10). That unit is mounted onto the rod (7) of the machine frame (9). The end pin of the rotor bearing (1) is driven by the flat belt (4). The driving flat belt (4) is tensioned by the idler pulley (6), which is damped by the pressure plate (5).

**Figure 3:**
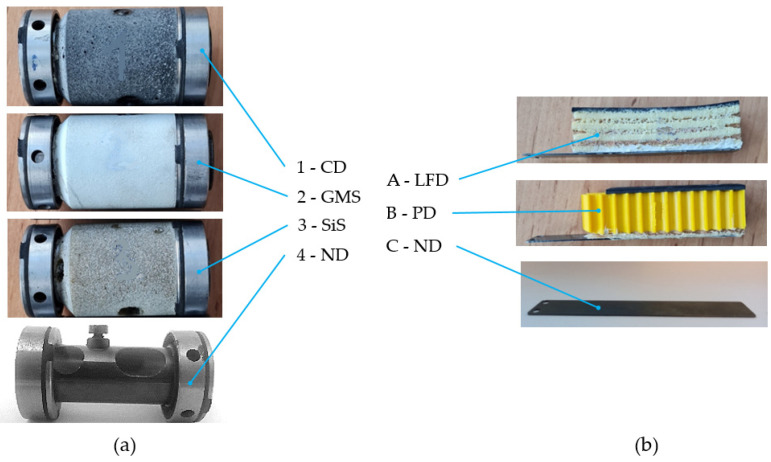
(**a**) Rotor casing filled with polymer matrix composites with 1-CD, carbon dust; 2-GMS, glass hollow microsphere; 3-SiS, silica sand; and 4-ND, native design (**b**) Pressure plate with A-LFD, layered foam damper; B-PD, particle damper; and C-ND, native design.

The native design (ND) of the rotor casing (2) (Figure 3a, 4-ND) consists of the main central thin-walled tube and two hollow steel rings on both ends of that tube. The screw secures the rotor against the axial motion. The native design of the pressure plate (Figure 3b, C-ND) is a sheet of steel plate of dimensions 20 × 110 mm of thickness 1 mm, with two holes for mounting at the one end of the plate. The purpose of that plate (5) is to damp the vibrations of the driving flat belt (4) before it comes into contact with the pin of the rotor bearing (1).

The new design of the rotor-bearing casing (Figure 3a) involves polymer matrix composites with different fillers: carbon dust 1-CD, glass hollow microsphere 2-GMS and silica sand 3-SiS. Those polymer matrix composites can be recognized as polymer concretes, whereas the fillers (aggregates) are bound together in a polymer matrix to create a composite material. The original pressure plate (Figure 3b) was supplemented by passive dampers based on the layered foam structural composite A-LFD (layered foam damper) and the structure for particle damping B-PD (particle damper). The implemented individual materials are described in the Materials subsection.

A measurement was made for each rotor casing with each pressure plate excited by the same rotor and same driving flat belt, with vibration amplitude excitation up to 5g. The range of tested speeds was 70,000–135,000 min^−1^ (1167–2250 Hz). A total of 12 combinations were included (Figure 2), each repeated 3 times.

### 2.1. Rotor Bearing

The flat belt drives the rotor bearing. While operating, the mutual interaction of other components of the mechanical system naturally occurs. Transverse vibrations of the belt cause fluctuations in the pressing force of the flat belt driving the rotor pin, and in this way, the deceleration and acceleration of its revolutions and undesirable torsional oscillations occur in the rotor. That force causes reaction forces inside the rotor bearing. Moreover, the forces acting inside the rotor are forces resulting from the kinematic of the bearing. When rotating, the rolling elements (balls) are pressed on the outer raceway surface. Loading forces are varied during operation with high frequency.

Naturally, the performance of the rotor bearing is related to its assembling and the manufacturing accuracy in terms of geometry and the dimensions of bearing components. The accuracy in the case of the tested rotor bearings is high, e.g., roughness *Ra* 0.02 μm, waviness 0.05 μm, and roundness 0.16 μm. This can be compared with a standard ball bearing, for which the roughness *Ra* is 0.08 μm, waviness is 0.5 μm, and roundness is 1 μm. Thus, the precision of the high-speed rotor bearing is 4–10 times higher. Before the rotor operation, the very precise dynamic balancing of the rotor is needed to meet the strict requirement of prescribed unbalance, i.e., 0.02 gmm (gram millimetres—unit of unbalance).

**Figure 4:**
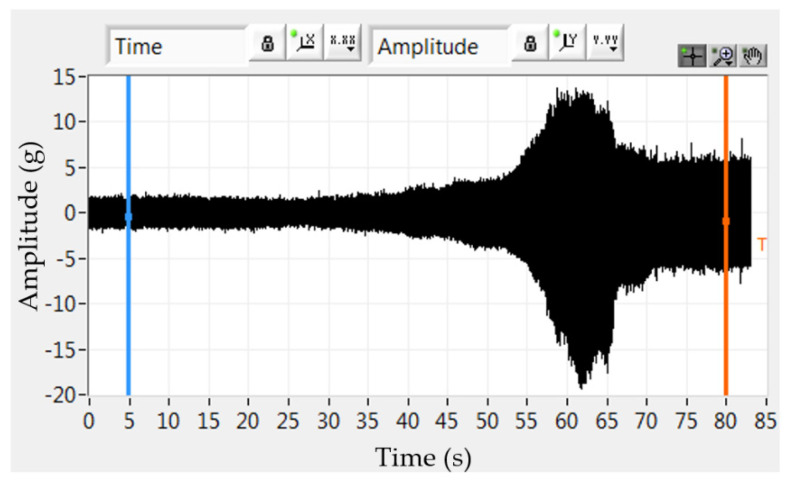
Time recording of acceleration amplitudes.

The typical response of the rotor bearing while continuously increasing the revolutions from 70,000 to 140,000 min^−1^ over 83 s is shown in Figure 4. The resonance peak was 127,000 min^−1^ in time 62 s.

### 2.2. Materials

The native design used the standard design materials, steel (rotor bearing, rotor casing, pressure plate, idler pulley) and aluminium alloy (housing body). Ceramic and plastic were used for the rolling elements and cage of the rotor bearing. The flexible casings at both ends of the rotor casing were hollow steel rings filled with rubber.

The supplemented additional materials were according to Table 1. The original weight of the pressure plate without a passive damper was 19.7 g, and the rotor casing without additional composites was 92 g.

**Figure 5:**
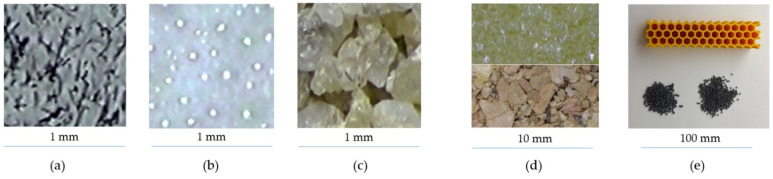
(**a**) Carbon dust, (**b**) glass hollow microspheres, (**c**) silica sand, (**d**) polymer foam (upper), cork (lower), (**e**) plastic honeycomb (upper) and lead particles (lower).

The fillers of the epoxy resin matrix were different types of material, i.e., carbon, glass and silica, of different grain sizes and shapes (photos provided in Figure 5a–c) with different weights (Table 1). Figure 5d shows the structure of both materials of layered foam damper A-LFD. Polymer foam (Figure 5d, upper) is made up of macroscopic open pores, while the structure of cork is naturally foamed at the microscopic level. Passive damper B-PD consisted of a honeycomb plastic base structure, freely filled with a mix of lead spherical particles of two sizes (Table 1, Figure 5e).

## 3. Results

Under extreme operating conditions, up to 135,000 min^−1^, the new design of the two components involving the additional materials manifested differences mainly in the amplitude values in the resonance peak. The volume of additional materials was the same for each component; however, damping, mass and stiffness were different. The additional materials, mainly polymer matrix composites, were the main sources of various dynamic responses documented in this paper.

The presented experimental study was preceded by particular prior research measurements and experiments of various modifications in design: (1) the various types of rubber in the end cavities of the rotor casing, (2) increasing the wall thickness of the steel tube of the rotor casing, (3) replacement of aluminium alloy in the housing body with cast iron, (4) the use of viscoelastic polymer damping material in the cavities of the housing body, (5) increasing the wall thickness of the ribs of the housing body, and (6) design changes in the rotor bearing itself (material, shape modifications). The mentioned modifications brought favourable results with varying levels of dynamic behaviour effect, manifested by a decrease in vibration amplitude and also acoustic emission, and subsequently, the possibility of a rotational speed increase. However, the use of polymer particle composites and non-standard materials added to the components of the native design is the research object of the presented experimental study with the goal to achieve a higher rotational speed, while keeping the amplitudes in a safe mode.

The amplitude reduction relative to the undamped structure is calculated as follows:

(6) $$A=\left(1-\frac{A_{\mathrm{damped}}}{A_{\mathrm{undamped}}}\right)\cdot 100\%$$

where *A*_damped_ and *A*_undamped_ are the amplitude of the damped and undamped structures, respectively.

**Figure 6:**
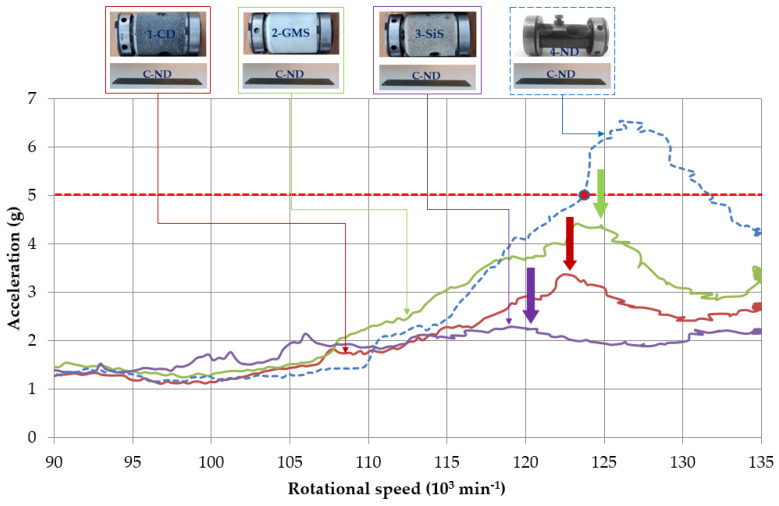
Dynamic responses of rotor housing design modification effect (continuous) and comparison with native design (dashed).

**Figure 7:**
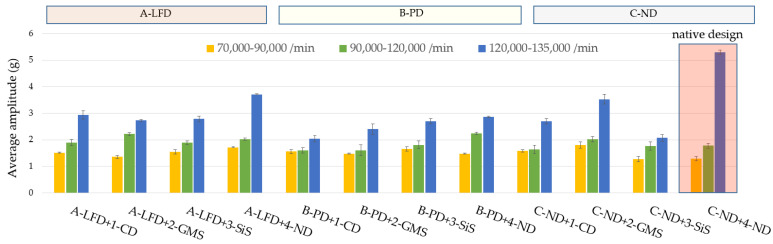
Average amplitudes comparison of pairs of the modified rotor casing and pressure plate.

The original design of 4-ND+C-ND without any additional composite materials is represented by a dashed blue curve in Figure 6 with a maximum amplitude of 6.5g (symbol g is the acceleration of gravity 9.8 m·s^−2^) at 126,000 min^−1^. The addition of three different particle composites with polymer matrix represented the change in the dynamic response of the machine system in the form of the amplitude reduction by 45.3%, 32.1%, and 60.4% in the speed range of 120,000–135,000 min^−1^ (Figure 6 and Figure 7, Table 2) for the polymer composite with carbon dust 1-CD (red in Figure 6), glass hollow microspheres 2-GMS (green in Figure 6), and silica sand 3-SiS (purple in Figure 6), respectively. In the case of the polymer composite filled with silica sand 3-SiS, the resonance peak decreased the most by 79.2% of its height and its peak is slightly visible. The above compared influence of the rotor housing modification by the inclusion of polymer particle composites significantly reduced the response in the form of a significant reduction in vibration amplitudes. Thus, the goal of increasing the rotational speed, while keeping the amplitudes under 5g, was achieved. Each modified resonance curve is lower and less sharp, and the resonance peak is reduced to safe levels. While the original design allowed safe operation up to about 124,000 min^−1^, the mechanical machine system with additional composite materials acting as passive dampers can operate at rotational speeds up to 135,000 min^−1^, with the possibility of higher speeds. The rotational speed increase was 8.9%; however, this could be higher as the measurements were taken up to 135,000 min^−1^. It should be noticed that the currently used operational revolutions in the production are 105,000–110,000 min^−1^.

According to the vibration theory, the higher mass of the rotor casing caused by additional material shifted the resonance peaks slightly to the left (green, red, and purple in Figure 6). Recall, in the basis of the vibration theory, the only way to reduce the resonance peak is due to the increased damping. The significant reduction in the amplitudes in the resonance peak was the result of significant damping, due to the high material damping of the implemented polymer composites with individual fillers, which is in accordance with the principles of the machine tool dynamics.

The disadvantage of the polymer composite filled with silica sand 3-SiS with the best result of the re-design in Figure 6 was a large weight, and we needed to find a lightweight solution as the rotor housing is attached to the rod of the frame that is not stiff enough for increased weight. We can consider the increasing damping of the pressure plate. Hence, another change in the original design of the machine mechanical system was the incorporation of passive dampers on the pressure plate, i.e., A-LFD consisting of layered foams and B-PD involving the particle damping principles. The results of average amplitudes in three ranges of rotational speeds of all pairs of the modified rotor casing and pressure plate are shown in Figure 7.

**Figure 8:**
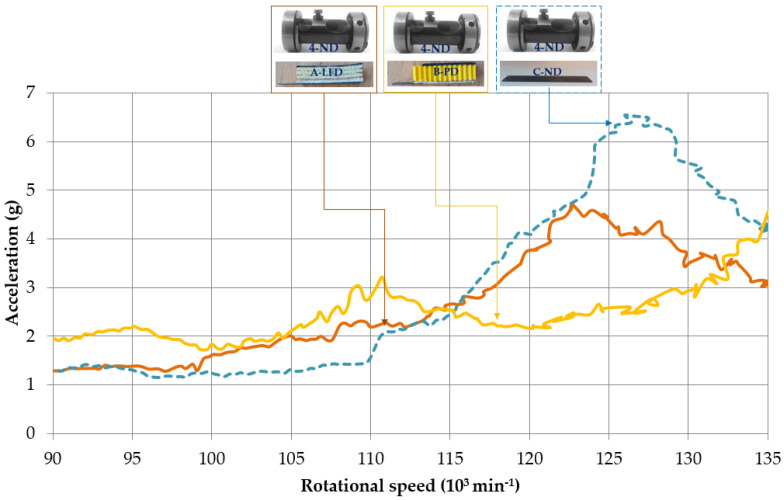
Dynamic responses of passive dampers with native rotor casings.

Applying the passive dampers to native rotor casings, the following reduction of vibration amplitudes was achieved (Figure 7 and Figure 8, Table 2): The amplitude reduction by 30.2% and 41.5% in a range of 120,000 to 135,000 min^−1^ for passive damper A-LFD and B-PD, respectively. A pressure plate with a passive damper A-LFD and original rotor casing 4-ND was able to reduce the maximum amplitude to 4.7g at 122,700 min^−1^ (brown in Figure 8). That maximum amplitude value was in safe operation mode as it was below 5g. The presence of the passive damper A-LFD and native rotor casing 4-ND enabled the reduction of the average amplitude to 3.7g in the abovementioned range (Figure 7). Recall that the original maximum amplitude of 6.5g and the average amplitude of 5.3g of C-ND+4-ND were in warning mode. The addition of passive damper B-PD using native rotor casing 4-ND caused the amplitude of 2.6g, i.e., a drop in that amplitude by 59.2% in a rotational speed of 126,000 min^−1^, which is a rotational speed for the original maximum amplitude 6.5g of native design C-ND+4-ND.

Incorporating passive dampers and individual polymer particle composites in the rotor casing, the following results were obtained: A-LFD+1-CD, A-LFD+2-GSM, and A-LFD+3-SiS provided a reduction in average and maximum amplitudes (Figure 7). Compared to the native design C-ND+4-ND, the reduction in the average amplitude values in the range of 120,000–135,000 min^−1^ was by 43.4%, 49.1%, and 47.2% (Table 2) for A-LFD+1-CD, A-LFD+2-GSM, and A-LFD+3-SiS, respectively. The original resonance peak was in the warning mode and the additional material in the form of a layered foam damper A-LFD decreased this in safe operation mode.

Even in the case of implementing a pressure plate B-PD and polymer particle composites within the rotor casing, there was a significant decrease in average amplitudes in the range of 120,000–135,000 min^−1^ by 69.8%, 50.9%, and 34.0% (Table 2) for B-PD+1-CD, B-PD+2-GSM, and B-PD+3-SiS, respectively. The implementation of a passive damper, A-LFD and B-PD and individual modified rotor bearings allowed an increase in the rotational speed by at least 8.9% (a higher rotational speed than 135,000 min^−1^ was not within the scope of measurement).

Evaluating the average contribution of individual additional materials, the resonance region of 120,000–135,000 min^−1^ showed significant changes in the dynamic response mainly by the reduction in the undesirable average amplitude of 5.3g of the native design of the rotor casing and the pressure plate 4-ND+C-ND. The polymer composite with carbon dust 1-CD in pair with A-LFD, B-PD and C-ND was able to reduce the average amplitudes values by 43.4%, 69.8, and 45.3% (Table 2, numbers of *A*), respectively, i.e., by 67.3% on average, followed by a reduction of 44% and 47.2% on average by the composites with glass microsphere 2-GMS and silica sand 3-SiS, respectively. Evaluating the passive dampers on the pressure plate, the highest amplitude reduction was achieved by the passive damper using particle damping B-PD by 49.1% on average (69.8 + 50.9 + 34.0 + 41.5/4, numbers of *A* from Table 2). Layered foam damper A-LFD reduced that amplitude by 42.5% on average.

In general, in regions up to the resonance peak, the amplitudes decrease due to the higher stiffness. The reduction by 15%, 3.5%, and 8.5% on average was measured in the region of 90,000 to 120,000 min^−1^ for 1-CD, 2-GMS, and 3-SiS for both damped and undamped pressure plates. In the region of 70,000 to 90,000 min^−1^, the influence of increased damping capacity of mechanical machine systems did not notably appear.

In the machine tool design, there is a general requirement to use the lightweight materials regarding low energy consuming while starting and braking components made of that materials; moreover, the forces of inertia are low. As described above, all added materials had an important effect on the decrease in amplitudes. However, the weight of the added mass was not the same (see Table 2). The measured results were evaluated with respect to the mass of the added material using the coefficient *E*_a_, i.e., the amplitude reduction efficiency. The values of the amplitude reduction efficiency *E*_a_ in Table 2 were evaluated according to the following formula [14]:

(7) $$E_{a}=\frac{A}{m_{a}}$$

where *A* is the amplitude reduction relative to the undamped structure and *m*_a_ is the additional mass of the damper as a proportion of the native mass of the structure calculated as

(8) $$m_{a}=\frac{m_{\mathrm{additional}\;\mathrm{material}}}{m_{\mathrm{pressure}\;\mathrm{plate}}+m_{\mathrm{rotor}\;\mathrm{casing}}}\cdot 100\%$$

where *m*_additional material_ is the mass of added material (polymer composite, layer foam material, and particle damper), *m*_rotor casing_ and *m*_pressure plate_ is the mass of the native design of rotor casing and pressure plate, respectively.

Considering the additional mass as a proportion of native mass, the best amplitude reduction efficiency *E*_a_ was for passive damper A-LFD and the native rotor casing 4-ND (*E*_a_ = 2.108); however, the amplitude reduction was the lowest, i.e., 30.2%. Considering both the amplitude reduction and the additional mass, the best result was for the rotor casing with polymer composite filled with glass hollow microspheres 2-GSM and the pressure plate with the layered foam damper A-LFD (*E*_a_ = 1.077) corresponding with 49.1% amplitude reduction. Considering the additional mass of passive dampers, the pressure plate with A-LFD had an amplitude reduction efficiency *E*_a_ 7.4 times greater than that of B-PD. The pressure plate with A-LFD was only 16 g, compared to the 163.6 g added weight of the B-PD pressure plate. These were excellent results of the A-LFD pressure plate made of foam materials. Regardless of the weight, the results were better for the B-PD pressure plate using particle damping.

**Figure 9:**
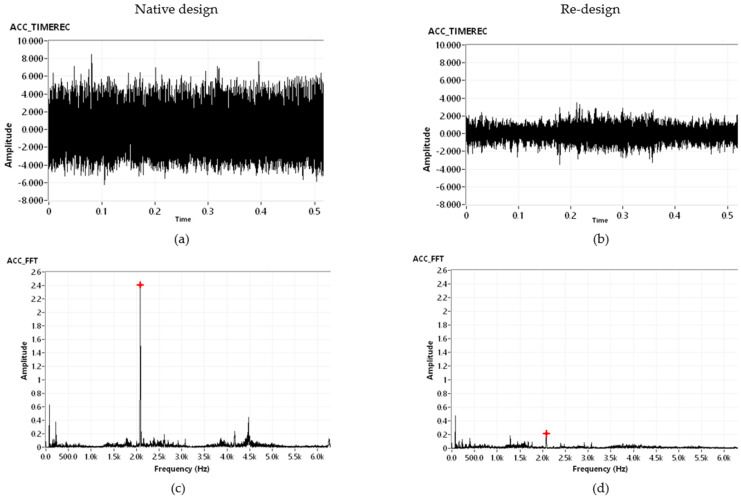
Comparison of time recording of (**a**) native design and (**b**) re-design, and comparison of FFT spectrum of (**c**) native design and (**d**) re-design.

Finally, the comparison of the amplitude reduction *A* of pairs of the modified components (Table 2) clearly showed the highest amplitude reduction *A* for the polymer composite with carbon dust 1-CD in the rotor casing and the passive damper using particle damping B-PD. A comparison of time and frequency domains of native design with the re-design dynamic response of B-PD+1-CD is illustrated in Figure 9. In that case, the average amplitude reduction was the largest, i.e., 69.8% (Figure 9a,b), and the amplitude of the fast Fourier transformation (FFT) spectrum (Figure 9c,d) was 91.3% lower; however, due to a large portion of additional mass, the amplitude reduction efficiency was 0.368.

## 4. Discussion

The damping properties of polymer composites are known, but their unsatisfactory mechanical properties make them of little use in the mechanical engineering industry, where the dominant structure materials are steel, cast iron or aluminium alloys of very low damping properties. Further, the reduction of vibration amplitudes is a serious problem as mechanical vibrations are a natural part, especially unwanted ones, which subsequently create sound, in any mechanical device while operating. This study shows the method how to combine the excellent mechanical properties of steel and the excellent damping properties of polymer composites. Thus, the gap between polymers and their applications in mechanical engineering can be filled.

In addition, the presented approach uses a re-design, which consists of supplementing the mechanical system by introducing the additional high-damping material on selected principal components of the mechanical system operating in a dangerous resonance region. The high-damping material is added in the form of attachment to a component or in the form of the filling of component cavities if they exist. A huge advantage of this approach is that there is no need to make a costly change to the original “steel” design to avoid resonance by adjusting the shape and dimensions of the component.

**Figure 10:**
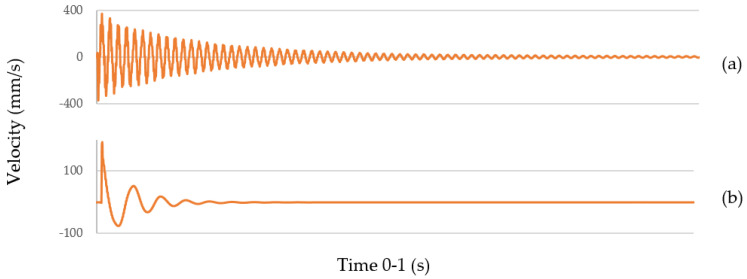
Time decaying curves of (**a**) steel and (**b**) polymer particle composite.

It is possible to compare the dynamic response of steel and polymer particle composite with silica sand in Figure 10, showing the free-damped vibration response in time (more in [34]). Comparing the steel dynamic response (Figure 10a), and the polymer composite (Figure 10b), logarithmic decrement is higher, the decay time is shorter, and the natural frequency is lower. It predetermines the use of polymer particle composites for applications with the need for high-vibration energy dissipation.

When testing in the given speed range, mainly radial vibrations are induced. However, due to the kinematical sliding of the rolling elements at extreme rotational speed, the unfavorable torsional oscillations can also be generated. The high-damping additive material in various tested combinations also changed the natural frequency of the components and mechanical system. Since the natural frequency of the system changed, the different combinations of materials reduced vibrations differently and the torsional vibrations were evoked at different rotational speeds. The object of future research in that mechanical system will be the sources of torsional vibrations and dependence on the forcing rotational speed and additional materials.

**Figure 11:**
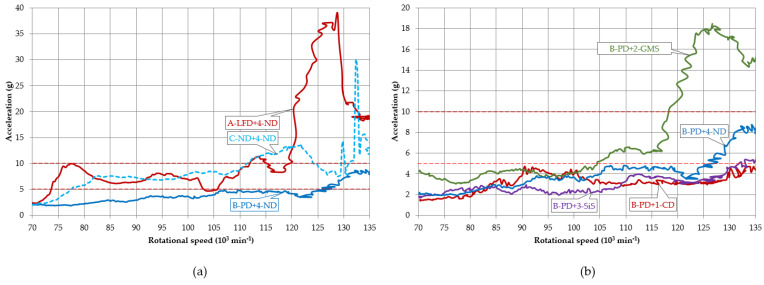
Dynamic response for generated vibration amplitudes above 5g (**a**) pressure plate, (**b**) rotor casing.

The tested amplitudes in the presented paper are up to 5g; however, while operating, the state of the rotor bearing can be changed by degenerative processes inside it and the vibration amplitudes can appear above 5g, which corresponds with the warning and danger operational mode. While passive damper A-LFP is not able to damp those very high amplitudes (Figure 11a), the passive damper B-PD is reliable even for very extreme conditions and its average amplitude is 4g in the range 70,000–135,000 min^−1^, while the original amplitudes of native design C-ND+4-ND are 8g.

In very large amplitudes, more than 5g, in the range 70,000–135,000 min^−1^ (Figure 11b), the average amplitudes for the polymer particle composites with carbon dust 1-CD and silica sand 3-SiS are 3.2g and 2.7g, respectively. The mentioned polymer particle composites in pair with passive damper B-PD are of excellent vibration amplitude reduction ability even for vibrations generated by a rotor above 5g.

The best combination of additional high-damped materials incorporated in the rotor casing and pressure plate was evaluated. The largest average amplitude reduction of 69.8% in the time domain and the maximum amplitude reduction of 91.3% in the frequency domain were with a combination of the rotor casing filled with a polymer composite with carbon dust, 1-CD, and the pressure plate filled with damper using particle damping, B-PD in resonance range 120,000–135,000 min^−1^. The mentioned B-PD+1-CD provides excellent dynamic behaviour, even in the case of the vibrations generated by the rotor above 5g. Considering the weight, the best amplitude reduction efficiency was for additional polymer composite with glass hollow microsphere 2-GMS at the rotor casing, and layered foam damper A-LFD at the pressure plate in the resonance range 120,000–135,000 min^−1^. The amplitude decrease was 49.1%. However, that pair is not able to sufficiently reduce vibrations above 5g. Furthermore, an important reduction of 60.4% was measured using only polymer composite with silica sand 3-SiS with the native pressure plate C-ND. The B-PD+3-SiS showed a very notable reduced dynamic response into safe operational mode even for vibrations generated by rotor above 5g.

## 5. Conclusions

This paper provides an experimental study with the aim of achieving an increase in rotational speed, keeping the vibration amplitudes within the safe operational mode in the resonance region. The resonance phenomenon is well known for its rapid increase in vibration amplitudes, even above the warning or danger limits. We made an application for high-speed rotating machinery with a current rotational speed of 105,000–110,000 min^−1^. This rotational speed was increased up to 135,000 min^−1^ with the possibility of an even higher speed while keeping the values of amplitudes below the warning limit. This speed increase is possible when applying a passive damper based on a layered composite structure, and with particle damping and filling of a structural cavity with polymer particle composites filled with carbon dust, glass hollow microspheres and silica sand, i.e., polymer concretes. Thus, a deep major structural design dimension modification is not needed. The resonance region is the damping control region, and the mentioned additional materials introduce high damping into the mechanical machine system, resulting in a decrease in the unwanted amplitudes of vibrations.

The resonance region of 120,000–135,000 min^−1^ showed significant changes in the dynamic response manifesting in the important vibration amplitude reduction in the range from 30 to 70% of the average vibration amplitude compared with the native design of induced vibration amplitudes up to and above 5g. The rotational speed can be increased up to about 20% compared with the currently used one.

## Figures and Tables

**Table 1:** Parameters of additional materials.

**Pressure Plate**	**A-LFD**	**B-PD**	
Material	Rigid polymer foam/cork foam: seven layers	Lead particles/plastic honeycomb	
Grain size (mm)	-	1.85 and 1.4 1:1 (volume ratio)	
Additional mass (g—gram)	16	163.6	
**Rotor casing**	**1-CD**	**2-GMS**	**3-SiS**
Material of filler	Carbon	Glass	Silica
Polymer composite filler	Dust	Hollow microspheres	Sand
Grain size (mm)	-	0.089 (average)	0.3–0.6
Weight ratio filler/matrix	0.18:1	0.06:1	2.3:1
Filler weight % (wt %)	15.1	6.3	70.0
Volume ratio filler/matrix	0.5:1	0.5:1	1.5:1
Filler volume % (vol %)	33.3	33.3	60.0
Additional mass (g—gram)	48.2	34.9	67.2

**Table 2:** Comparison in resonance region through *E*_a_.

Pairs	Average Amplitude (g)		*A* (%)	Additional Mass (g—gram)	*m*_a_ ** (%)	*E*_a_ (-)
	Damped	Undamped *				
C-ND+1-CD	2.9	5.3	45.3	48.2	43.2	**1.049**
C-ND+2-GMS	3.6	5.3	32.1	34.9	31.2	1.027
C-ND+3-SiS	2.1	5.3	60.4	67.5	60.4	0.999
A-LFD+1-CD	3.0	5.3	43.4	64.2	57.5	0.755
A-LFD+2-GMS	2.7	5.3	49.1	50.9	45.6	**1.077**
A-LFD+3-SiS	2.8	5.3	47.2	83.5	74.8	0.631
A-LFD+4-ND	3.7	5.3	30.2	16.0	14.3	**2.108**
B-PD+1-CD	1.6	5.3	**69.8**	211.8	189.6	0.368
B-PD+2-GSM	2.6	5.3	50.9	198.5	177.7	0.286
B-PD+3-SiS	3.5	5.3	34.0	231.1	206.9	0.164
B-PD+4-ND	3.1	5.3	41.5	163.6	146.5	0.283

* amplitude of machine system without any additional material; ** native mass of rotor casing and pressure plate is (92 + 19.7) = 111.7 gram.

## Data Availability

Not applicable.
